# Antidepressant effects of total iridoids of *Valeriana jatamansi* via the intestinal flora-blood–brain barrier pathway

**DOI:** 10.1080/13880209.2021.1944222

**Published:** 2021-07-08

**Authors:** Li Zhang, Liwen Wang, Li Huang, Yanni Zhao, Hongling Ding, Binglong Li, Lingmiao Wen, Wei Xiong, Yanjun Liu, Tinglan Zhang, Liudai Zhang, Lanlan Wu, Qing Xu, Yuqing Fan, Guihua Wei, Qiaozhi Yin, Yunhui Chen, Tiane Zhang, Zhiyong Yan

**Affiliations:** a School of Life Science and Engineering, Southwest Jiaotong University, Chengdu, PR China; b School of Basic Medicine, Chengdu University of Traditional Chinese Medicine, Chengdu, PR China

**Keywords:** Depression, blood–brain barrier (BBB) permeability, firmicutes, lactobacillus, bacteroidetes, functional prediction

## Abstract

**Context:**

*Valeriana jatamansi* Jones [syn. *V. wallichii* DC, (Valerianaceae)] (VJJ) is used to treat depression.

**Objective:**

To explore the effects of total iridoids of VJJ extract (TIV) on chronic unpredictable mild stress (CUMS) in mice.

**Materials and methods:**

VJJ roots and rhizomes were extracted with 70% ethanol. CUMS rats were treated daily with fluoxetine (2.6 mg/kg, i.g.) or TIV (5.7, 11.4, and 22.8 mg/kg, i.g.) for 14 days. Male Kun Ming mice on normal chow and 0.5% CMC–Na solution were used as a control. Behavioural tests included the tail suspension (TST) and sucrose preference tests (SPT). Evans blue staining was used to evaluate blood–brain barrier (BBB) permeability. Western blotting was used to measure zonula occludens-1 (ZO-1) and occludin expression. 16S rRNA sequencing was used to analyse intestinal flora abundance. Tax4Fun was used to predict KEGG metabolic pathways.

**Results:**

TIV treatment reduced TST time (117.35 ± 8.23 or 108.95 ± 6.76 vs. 144.45 ± 10.30 s), increased SPT (55.83 ± 7.24 or 53.12 ± 13.85 vs. 38.98 ± 5.43%), increased the abundance of phylum Firmicutes (86.99 ± 0.03 vs. 60.88 ± 0.19%) and genus *Lactobacillus* (75.20 ± 0.19 vs. 62.10 ± 0.13%), reduced the abundance of phylum Bacteroidetes (6.69 ± 0.06 or 11.50 ± 0.09 vs. 25.07 ± 0.20%). TIV increased carbohydrate metabolism (14.50 ± 3.00 × 10^−3^ or 14.60 ± 2.00 × 10^−3^ or 14.90 ± 2.00 × 10^−3^ vs.13.80 ± 4.00 × 10^−3^%), replication and repair functions (5.60 ± 1.00 × 10^−3^ or 5.60 ± 1.00 × 10^−3^ vs. 5.10 ± 4.00 × 10^−3^%), reduced the frequency of infectious disease (1.60 ± 2.00 × 10^−4^ or 1.90 ± 5.00 × 10^−4^ or 1.80 ± 3.00 × 10^−4^ vs. 2.20 ± 7.00 × 10^−3^%), BBB permeability (0.77 ± 0.30 vs. 1.81 ± 0.33 μg/g), and up-regulated the expression of ZO-1 (1.42-fold, 1.60-fold, 1.71-fold) and occludin (1.79-fold, 2.20-fold).

**Conclusions:**

TIV may modulate the intestinal flora, thereby inducing the expression of ZO-1 and occludin, protecting the BBB and exerting an antidepressant effect.

## Introduction

Depression, also known as depressive disorder, is a mental illness with clinical manifestations that include significant and persistent depression, retardation of thinking, depressed mood, sleep disorders, and lack of interest or excitement. Drug therapy is more widely used than psychotherapy in antidepressant therapy (Cipriani et al. 2018). Despite the wide variety of antidepressant drugs available, antidepressants are not efficacious in nearly 40% of patients at 6–12 weeks after beginning treatment, and more than 50% of patients are not cured (Gartlehner et al. 2012). Antidepressants have short clinical efficacy and varying degrees of adverse reactions. Hence, the study of antidepressants has attracted much attention, especially in light of the sharply increased incidence of depression.

The blood–brain barrier (BBB) is a specialised selective permeability barrier between the brain tissue and blood, also known as the brain’s ‘first line of defense’ (Kealy et al. 2020). Tight junctions (TJ) are crucial to ensuring the basic structure and function of the BBB and play a key role in membrane permeability (Di Marco et al. 2020). The gut microbiota also maintains BBB integrity by upregulating the expression of zonula occludens-1 (ZO-1) and claudin-5 (Braniste et al. 2014). Destruction of the BBB contributes to central nervous diseases, such as Alzheimer’s disease, Parkinson’s disease, and encephalitis (Tikiyani and Babu 2019). Changes in intestinal microbial diversity may cause disorders that eventually lead to depression, autism, and schizophrenia (Rook et al. 2013; Kigerl et al. 2016; Sherwin et al. 2016). The intestinal flora regulates gastrointestinal function and central nervous system (CNS) homeostasis via their effects on the intestinal flora-brain-gut axis (Cryan and Dinan 2015).

The roots and rhizomes of *Valeriana jatamansi* Jones [syn. *V. wallichii* DC, (Valerianaceae)] (VJJ) have been used for thousands of years as traditional Chinese medicine, first published in the Compendium of Materia (Li 1975). The roots and rhizomes of VJJ are medicinal, and their effects include promoting digestion (Khan and Gilani 2011), reducing diarrhoea (Dong et al. 2018), smoothing the circulation (Prasad 2010), eliminating pain, and tranquilising the mind (Sah et al. 2010). VJJ contains many chemical components, such as iridoids (Tang et al. 2002; Lin et al. 2009), flavonoids (Li 2009), alkaloids, and volatile oils (Letchamo et al. 2004; Singh et al. 2006; Tan 2019). Iridoids are the main active component of VJJ and have low toxic side effects (Xu et al. 2015). Water, methanol, ethanol, and patchouli extracts of VJJ show antidepressant activity (Subhan et al. 2010). In a recent study, TIV improved depressive disorder and visceral hypersensitivity by a mechanism related to increased 5-HT and NE and decreased gastrointestinal motility (Shi et al. 2011; Tao 2017). Our group has demonstrated that TIV is safe at clinical dosages and shows no single-dose toxicity in mice and rats. The lethal dose with 50% mortality rate (LD_50_) on mice is over 2000 mg/kg bw (Xu et al. 2015). We also showed that TIV exerts an antidepressive effect by regulating multiple metabolic pathways (Li et al. 2020) and regulates the intestinal flora in depressed mice (Wang et al. 2020). What remains unclear is how the intestinal flora and BBB are involved in the anti-depression activities of TIV. Here, we describe a mouse model of depression based on chronic unpredictable mild stress (CUMS). This is the first study to use this model to explore the effects of TIV on depression via the BBB–intestinal flora pathway. The results of this study provide the basis for further research on the antidepressant mechanisms of TIV.

## Materials and methods

### Chemicals and drugs

The chemicals and drugs used in this study include ethanol (Hengxin, China), 0.5% CMC–Na (Kelun, China), fluoxetine (Lilly, China, J20180001, Batch No. 7057948), 2% Evans blue (EB) (Sigma), 50% trichloroacetic acid (Lingang, China), and 3% pentobarbital sodium (Senbei Jia, China). Occludin (No. ab216327) and ZO-1(No. ab96587) were purchased from Abcam (United Kingdom). The BCA kit (Xavier Bio, No. XS184503), secondary antibody (Lianke, A90641), and CTAB kit (North Olebo, No. ALH603) were obtained from different companies in China.

### Plant collection and identification

The roots and rhizomes of VJJ were purchased from the Lotus Pond Chinese herbal medicine market in Chengdu, southwestern China, in April 2018. The samples were identified as VJJ by Professor Liangke Song from the School of Life Science and Engineering, Southwest Jiaotong University, China, where the sample (No. 20181003) was stored.

### TIV extraction and identification

Extraction was performed as described previously (Xu et al. 2014). The dry powder of VJJ (10.4 kg) was extracted three times with 70% ethanol (24 h each time). The ethanol extract (2.32 kg) was obtained by filtration and vacuum distillation. The extract was diluted with distilled water and sonicated. A large amount of water was used to disperse the alcohol extract, and the material was purified with D101 macroporous resin (Baoen, Cangzhou, China, No. 110901). The 95% ethanol eluent was collected and concentrated to obtain the oil substance referred to as TIV (0.19 kg; purity, 76.58% ± 2.14%; RSD, 2.8%). We established a quality control standard for TIV, and the chlorovaltrate and valjatrate B contents were 8.91 and 7.00 mg/g (Xu et al. 2015; Zhu et al. 2016). TIV was preserved at 4 °C without light.

### Experimental animals

A total of 72 adult male Kun Ming mice (SPF, weight: 16–20 g) provided by Dashuo Experimental Animal Co. Ltd. (Chengdu, China), with certificate number SCXK (Chuan) 2015-030, were used in this study. The mice were raised in plastic cages with a constant temperature of 23 °C, constant humidity, and a 12 h alternating light-dark cycle. The mice had free access to food and water until the beginning of the experiment. The experiments were approved by the animal ethics committee of Southwest Jiaotong University (No. S20190925003) and conducted according to the college’s animal experiment guidelines.

### Animal grouping and model establishment

After 1 week of adaptive feeding, the mice were divided into normal, model, fluoxetine, TIV-L (TIV-low dose), TIV-M (TIV-medium dose), and TIV-H (TIV-high dose) groups, with 12 mice per group. Mice in the normal group were group-housed, but all other groups were kept in solitary cages. The normal group was raised in a stress-free environment, while all other groups were randomly subjected to stress every day according to the CUMS modelling method described by Biala et al. (2017). Stresses included food-fast (food deprivation for 24 h), water-fast (without water for 24 h), damp bedding (soaked with water), bondage (mice were bound in a restraint tube for 2 h), cold water swimming (mice swam in 4 °C water for 5 min) and tail-clamping (1 min with tail-clamping forceps). The same stress was not given continuously, and the modelling lasted for 4 weeks. The mice were weighed before modelling, after modelling, and after drug administration.

### Administration regimen

Drugs were administered by gavage once a day for 14 days, beginning 2 weeks after the modelling stress began. Mice were randomly administered one stress, 1 h after treatment. The normal and model groups were given 0.5% CMC–Na, and the fluoxetine group was given 2.6 mg/kg/d fluoxetine dispersible tablet. The dosages in the TIV-L, TIV-M, and TIV-H groups were 5.7, 11.4, and 22.8 mg/kg/d, respectively.

### Behavioural tests

The purpose of the tail immobility time (tail suspension test, TST) was to verify the model and evaluate drug efficacy. TST was performed 4 weeks after modelling and 2 weeks after drug administration as described (Nakagawasai et al. 2020). Mice were hung upside down on the tail suspension instrument in a quiet environment. After the mice adapted to the instrument for 2 min, the tail suspension immobility time was recorded over a 4 min test period. Mouse activity was recorded using a digital camera.

The sugar solution consumption rate (sucrose preference test, SPT) was used to assess the mice for loss of pleasure. SPT was conducted as described (Tobiansky et al. 2020), 4 weeks after modelling and 2 weeks after drug administration. On the first day, two bottles of sucrose water (1% sucrose solution) were placed in each cage. On the second day, one bottle of sucrose water and one bottle of pure water were randomly placed in each cage, and the position of the water bottle was changed every 4 h to eliminate the influence of bottle position on the experiment. Then, the mice were deprived of water for 12 h. Each cage was given a weighed bottle of pure water and a bottle of sucrose water. The mice had free access to water for 12 h after the third day, and water bottle weights were recorded. The sucrose consumption rate was calculated as follows:

sucrose preference(%)=  sucrose consumption/(water consumption+sucrose consumption) × 100%.

### EB staining and electron microscopy

EB can stabilise albumin, but when the BBB structure is destroyed, it will enter the brain and stain the brain tissue (Gao et al. 2011). As described elsewhere (Deng et al. 2019), mice were injected with 2% EB solution (4 mL/kg) into the tail vein after behavioural tests. After 2 h, the mouse’s heart was rapidly perfused to remove EB from the blood. Then, the mouse’s cerebellum was removed and weighed. Finally, the brain tissue was homogenised in 50% trichloroacetic acid solution, and the supernatant was collected. Fluorescence intensity (Ex = 620 nm and Em = 680 nm) was measured in each sample using an enzyme labelling instrument (Jiangsu, China). EB dye quantity was calculated using a standard curve and expressed as mg/g brain tissue.

The TJ ultrastructure reflects the state of the BBB (Li et al. 2009). As described previously (Zhang et al. 2011), the mice were weighed and then anaesthetised deeply. Hearts were perfused with 0.9% normal saline (about 30 mL) and 3% pentobarbital sodium. After perfusion, the brain tissue was removed, fixed twice, dehydrated, infiltrated, and placed into a mould to form an embedded block. The embedded block was cut into 50 nm ultrathin sheets and double stained. The BBB structure was observed by transmission electron microscopy (JEM-1400PLUS).

### Western blot assay

Western blotting was used as described (Perrech et al. 2019) to measure ZO-1 and occludin protein levels. The brain tissue was homogenised on ice until fully lysed, and protein concentration was quantified using a BCA kit. Protein lysates (50 g) were separated by standard SDS-PAGE (Sevier Bio XS184503) and transferred to a PVDF membrane. The membrane was blocked for 90 min and then incubated with primary antibodies for occludin and ZO-1 overnight at 4 °C. The next day, membranes were incubated with a secondary antibody for 90 min at room temperature, then the image was developed. The optical density of each protein brand and the ratio of the optical density of each target protein to GAPDH were determined using software.

### Faeces handling and collection

Three mice were randomly selected from each group. The abdominal cavity was opened aseptically, and faeces were collected from the colon, then numbered and stored at –20 °C for later use.

### DNA extraction and PCR amplification

A kit (CTAB) was used to extract faecal DNA. DNA purity and concentration were measured by 0.8% agarose gel electrophoresis. Each sample was diluted to 1 ng/μL with sterile water and PCR-amplified with primers: [16S V4 region primers, 341 F (5′-CCTAYGGGRBGCASCAG-3′) and 806 R (5′-GGACTACHVGGGTWTCTAAT-3′)]. Electrophoresis on a 2% agarose gel was used to detect the amplification products, and the samples were mixed in equal amounts according to concentration. Finally, each PCR product was purified by agarose gel electrophoresis, and the target band was recovered by shearing.

### Library construction and sequencing

The V4 region of 16S rRNA was analysed by high-throughput screening and sequencing using the Rhonin’s MiSeq platform. The library was constructed using the TruSeq-free DNA sample kit, and the library was sequenced with the MiSeq platform PE300 (Thermo Fisher) after quantification and library testing.

### Functional prediction with Tax4Fun

The 16S rRNA sequencing data based on the SILVA database were clustered and annotated. The pre-calculated correlation matrix provided a linear conversion of the calculation results to obtain the classification spectrum of the microorganisms in the KEGG database. The results were corrected by comparison to the 16S rRNA gene sequences of different bacterial groups in NCBI. According to the classification information of the functional gene spectrum of microorganisms in the KEGG database, the results were linearly predicted.

### Data analysis

The experimental data were processed using the statistical software SPSS 21.0. The data were compared using *t*-tests, and results are expressed as mean ± standard deviation (x ± SD). Multiple samples were compared via one-way ANOVA, and the least significant difference test was used to compare groups. *p* < 0.05 was used to define statistical significance.

## Results

### Effect of TIV on weight and behaviour

**Figure 1.**
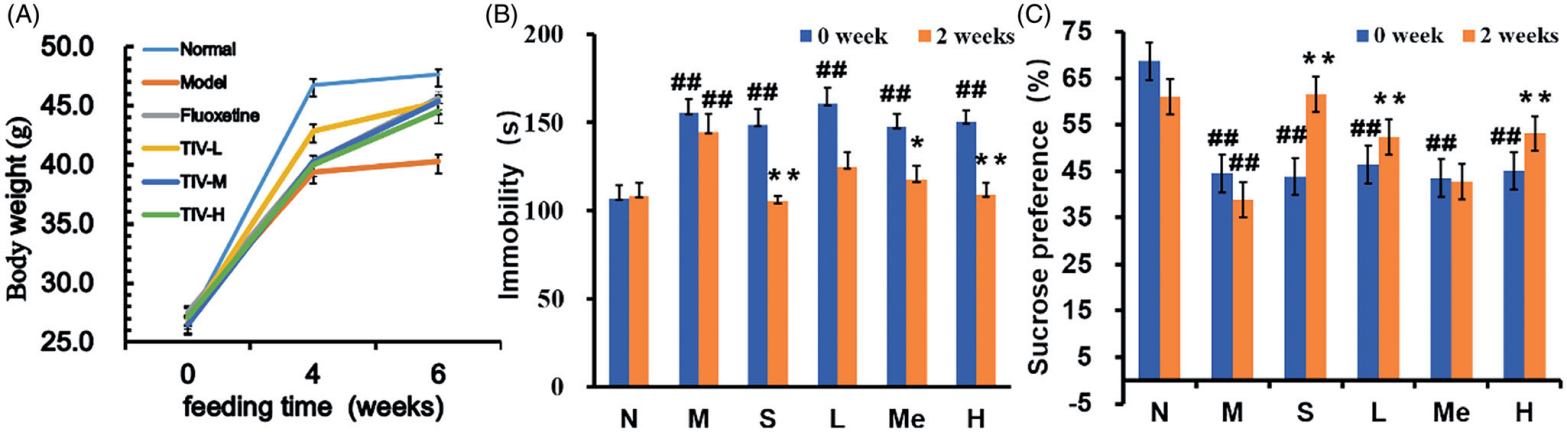
Behavioural evaluation of TIV-treated CUMS mice and controls. (A) Changes in body weight. (B) Immobility time in TST for CUMS mice with TIV treatment. (C) Sucrose preference in SPT for CUMS mice with TIV treatment. Data are reported as mean ± SD. # *p* < 0.05 and ## *p* < 0.01 vs. the normal group. *p < 0.05 and **p < 0.01 vs. the model group. N: normal; M: model; S: fluoxetine; L: TIV-L; Me: TIV-M; H: TIV-H.

The CUMS depression mouse model was established to explore the effect of TIV on weight and behaviour in depressed mice. There was no significant difference in weight before modelling (Figure 1(A)). After 4 weeks of modelling, the model group’s weight was significantly lower than that of the normal group (39.43 ± 2.10 vs. 47.35 ± 2.92 g, *p* < 0.01). The fluoxetine (45.86.10 ± 1.99 g, *p* < 0.01) and TIV (44.21 ± 1.42 g, *p* < 0.01; 44.57 ± 1.99 g, *p* < 0.01 and 43.57 ± 2.44 g, *p* < 0.01 for TIV-L, TIV-M, and TIV-H, respectively) treatment groups’ weight increased significantly compared to the model group. The TST of the CUMS model mice was significantly prolonged (Figure 1(B); 144.45 ± 10.30 vs. 106.03 ± 7.31 s, *p* < 0.01), and the SPT was significantly decreased (Figure 1(C); 38.98 ± 5.43 vs. 60.99 ± 5.97%, *p* < 0.01) (vs. the normal group). However, the tail suspension immobilisation times in the fluoxetine (105.15 ± 2.88 s, *p* < 0.01), TIV-M (117.35 ± 8.23 s, *p* < 0.05), and TIV-H (108.95 ± 6.76 s, *p* < 0.01) treatment groups decreased after drug administration. The sugar solution consumption rate in the TIV-L (55.83 ± 7.24%, *p* < 0.01) and TIV-H (53.12 ± 13.85%, *p* < 0.05) groups increased significantly compared to the CUMS model group. The altered behaviour of mice detected through weight diversity curves, TST and SPT, proved that TIV has an antidepressant effect.

### Effect of TIV on the intestinal flora

#### Sequencing quality of flora and distribution of OTUs

**Figure 2.**
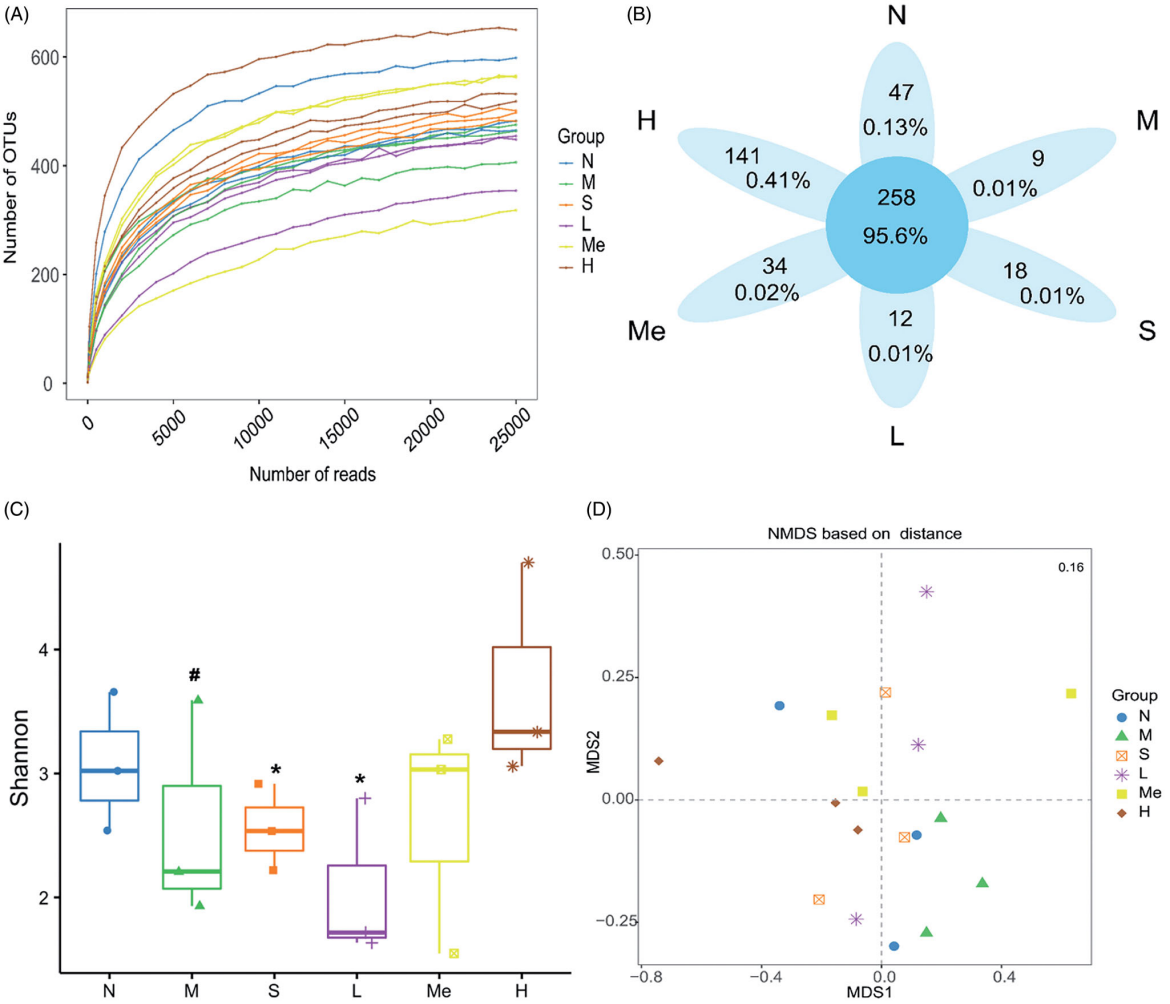
Reflectance curve (A) and Venn diagram (B) of intestinal microbial OTUs from TIV-treated CUMS mice and controls. (C) The alpha diversity of the Shannon index of intestinal microflora. (D) Analysis of principal coordinates based on Bray-Curtis distance (the stress values in the NMDS analysis of the differences between communities). Data are reported as mean ± SD. N: normal group; M: model; S: fluoxetine; L: TIV-L; Me: TIV-M; H: TIV-H (*n* = 3 per group).

As the depth of sequencing increases, the sample dilution curve gradually flattens (Figure 2(A)), indicating that the sequencing data covers all species in the sample. We obtained a total of 519 OTUs from six groups of sequencing data of intestinal flora. The Venn diagram compares the differences between OTUs. As indicated in Figure 2(B), six groups shared 258 OTUs. Unique OTUs were observed in the stress-free normal (47), CUMS model without treatment (9), fluoxetine (18), TIV-L (12), TIV-M (34), and TIV-H (141) groups.

#### Diversity analysis of the intestinal flora

The α diversity index was used to determine if significant taxonomic changes occurred. As shown in Figure 2(C), the model group exhibited a decrease in the alpha diversity of the Shannon index compared with the normal group (1.51 ± 0.27 vs. 3.07 ± 0.56, *p* < 0.05). TIV-L (3.35 ± 0.17, *p* < 0.05) and fluoxetine (3.05 ± 1.06, *p* < 0.05) treatment tended to increase the Shannon index (vs. the CUMS model group). NMDS (Non-Metric Multi-Dimensional Scaling) analysis based on OTU abundance was used to show a distinct clustering of gut microbiota compositions between treatments (Figure 2(D)). The stress value was 1.5, indicating that the NMDS analysis was reliable.

#### Intestinal flora abundance

**Figure 3.**
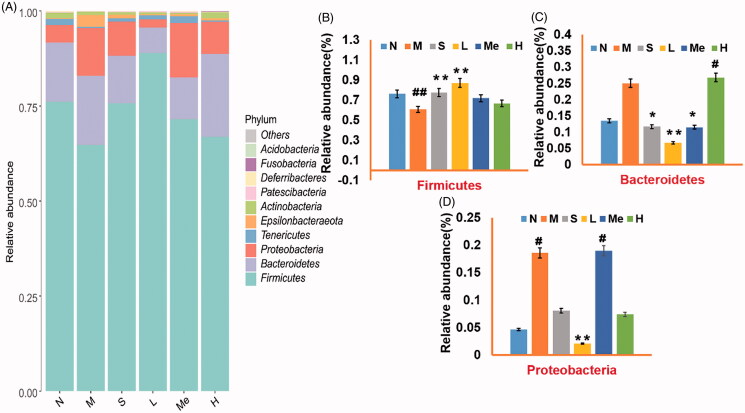
Comparison of gut microflora of (A) Barplot of relative abundance at the phylum level; the relative abundance of (B) Firmicutes, (C) Bacteroidetes, and (D) Proteobacteria. # *p* < 0.05 and ## *p* < 0.01 vs. the normal group. *p < 0.05 and **p < 0.01 vs. the model group. Data are reported as mean ± SD. N: normal; M: model; S: fluoxetine; L: TIV-L; Me: TIV-M; H: TIV-H (*n* = 3 per group).

The effects of CUMS on the composition and function of the intestinal flora were analysed via 16S rRNA sequencing. The community composition was analysed to predict the abundance and diversity of each species (Song et al. 2018). At the phylum level, Bacteroidetes, Firmicutes, and Proteobacteria predominated in all samples but varied in their abundances (Figure 3(A)). As shown in Figure 3(B–D), the number of Firmicutes (60.88 ± 0.19 vs. 76.11 ± 0.12%, *p* < 0.01) and Proteobacteria (18.62 ± 0.08 vs. 4.65 ± 0.06%, *p* < 0.05) in the CUMS model group changed relative to the normal group. The abundance of Firmicutes in the fluoxetine (77.49 ± 0.07%, *p* < 0.01) and TIV-L (86.99 ± 0.03%, *p* < 0.01) were significantly higher than that of the model group. Bacteroidetes in the fluoxetine (11.67 ± 0.08%, *p* < 0.05), TIV-L (6.69 ± 0.06%, *p* < 0.01) and TIV-M (11.50 ± 0.09%, *p* < 0.05) were lower than that of the model group (25.07 ± 0.20%). Proteobacteria in the TIV-L were less abundant than in the model group (2.10 ± 0.10 vs. 18.62 ± 0.08%, *p* < 0.01).

**Figure 4.**
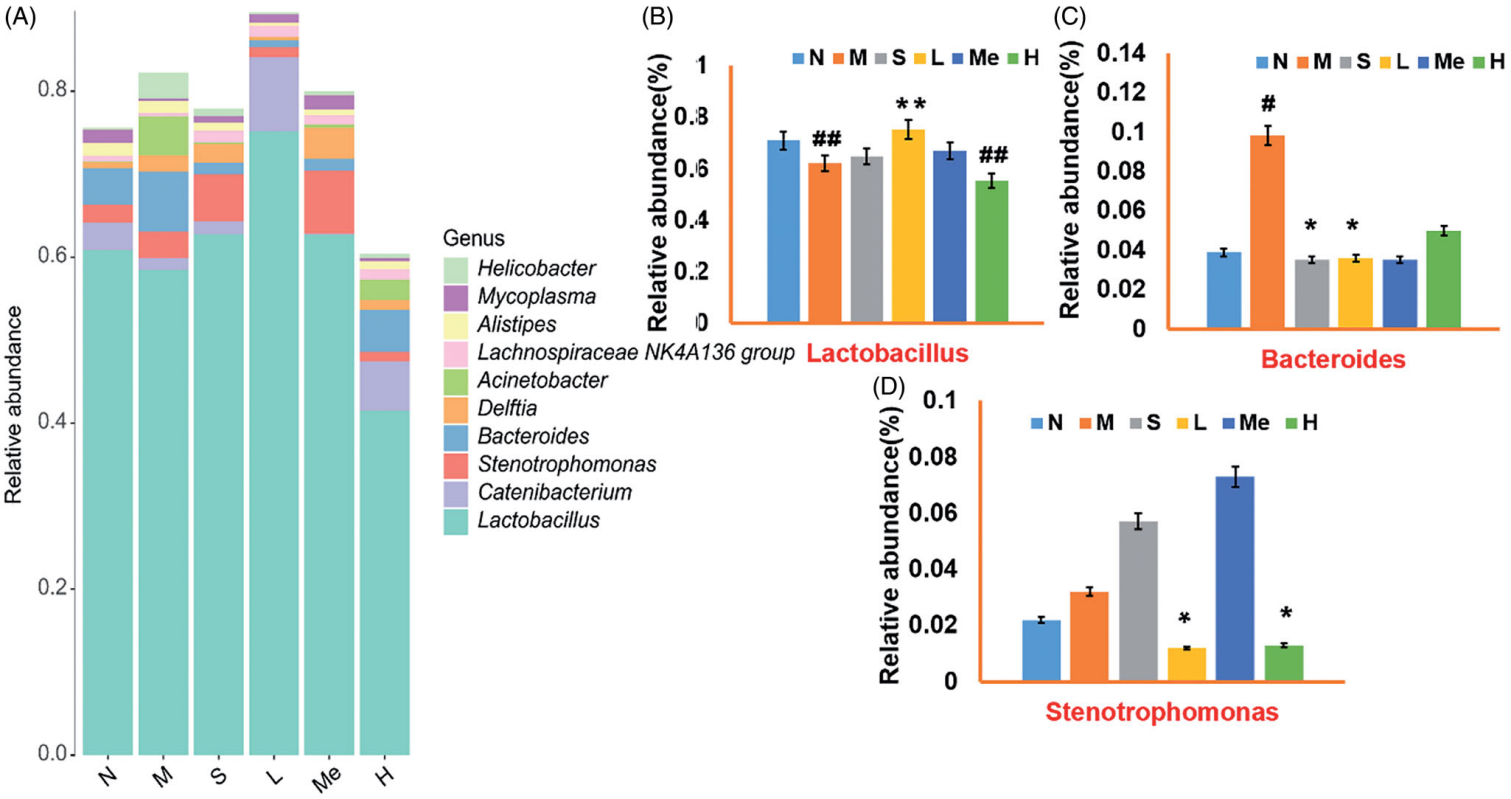
Comparison of microbial gene catalog of (A) Barplot of relative abundance at the genera level; the relative abundance of (B) *Lactobacillus*, (C) *Bacteroides*, and (D) *Stenotrophomonas*. # *p* < 0.05 and ## *p* < 0.01 vs. the normal group. *p < 0.05 and **p < 0.01 vs. the model group. Data are reported as mean ± SD. N: normal; M: model; S: fluoxetine; L: TIV-L; Me: TIV-M; H: TIV-H (*n* = 3 per group).

**Figure 5.**
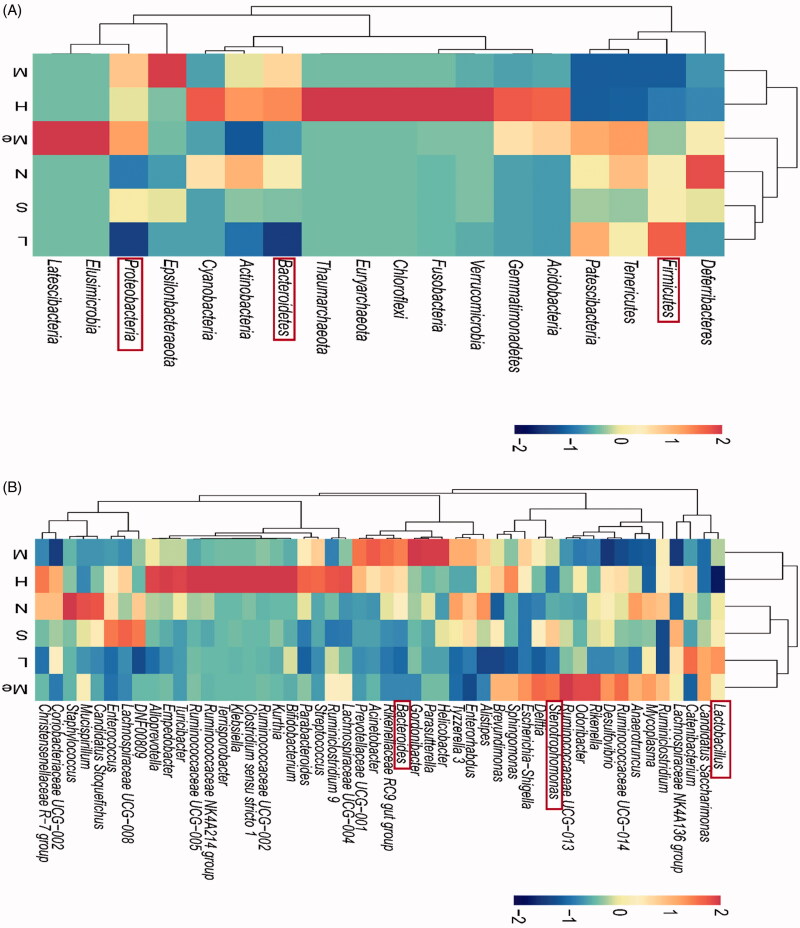
Heatmap of the relative abundances of (A) top 50 phylum and (B) top 50 genera. The red rectangle represents the relative abundance of the changed phylum and genera. # *p* < 0.05 and ## *p* < 0.01 vs. the normal group. *p < 0.05 and **p < 0.01 vs. the model group. Data are reported as mean ± SD. N: normal; M: model; S: fluoxetine; L: TIV-L; Me: TIV-M; H: TIV-H (*n* = 3 per group).

At the genera level, *Lactobacillus, Stenotrophomonas,* and *Bacteroides* were predominant in all samples (Figure 4(A)). Figure 4(B,C) indicate *Lactobacillus* was more prevalent in the TIV-L group (75.20 ± 0.19 vs. 62.10 ± 0.13%, *p* < 0.01) and *Bacteroides* in the fluoxetine (3.50 ± 0.03%, *p* < 0.05) and TIV-L group (3.60 ± 0.02%, *p* < 0.05) were least prevalent than in the model group (9.80 ± 0.04%). *Stenotrophomonas* decreased in the TIV-L (1.20 ± 0.01%, *p* < 0.05) and TIV-H group (1.30 ± 0.01%, *p* < 0.05) [vs. the CUMS model group (3.20 ± 0.04%)]. The relative abundances of the top 50 phylum and genera in faecal samples are shown in Figure 5(A,B). Compared with the model group, the three largest phyla were altered in the fluoxetine and TIV-L, including increased Firmicutes (i.e., *Lactobacillus* spp.) and decreased Bacteroidetes (i.e., *Bacteroides* spp.) and Proteobacteria (i.e., *Stenotrophomonas* spp.).

#### Metabolic function prediction of the altered gut microbiota

**Figure 6.**
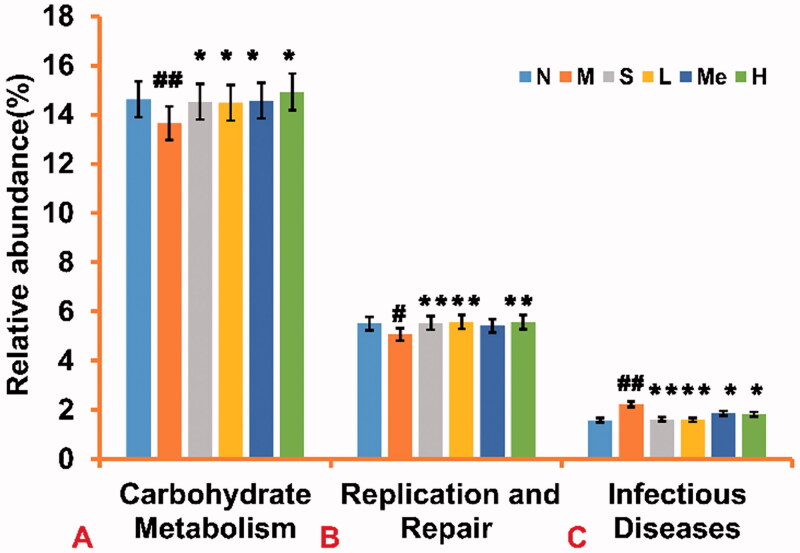
Comparison of metabolic function prediction of intestinal flora from TIV-treated CUMS mice and control group. (A) Relative abundance of carbohydrate metabolism. (B) Relative abundance of replication and repair functions. (C) Relative abundance of infectious diseases in mice. Data are reported as mean ± SD. # *p* < 0.05 and ## *p* < 0.01 vs. the normal group. *p < 0.05 and **p < 0.01 vs. the model group. N: normal; M: model; S: fluoxetine; L: TIV-L; Me: TIV-M; H: TIV-H (*n* = 3 per group).

A Tax4Fun analysis was performed to investigate microbiota function (Huang et al. 2020). The method provides an effective link between the OTU species classification annotated by the SILVA database and the prokaryotic metabolism function in the KEGG database. The relative abundance of carbohydrate metabolism (Figure 6(A)) was increased in the fluoxetine (14.50 ± 4.00 × 10^−3^%, *p* < 0.05), TIV-L (14.50 ± 3.00 × 10^−3^%, *p* < 0.05), TIV-M (14.60 ± 2.00 × 10^−3^%, *p* < 0.05) and TIV-H (14.90 ± 2.00 × 10^−3^%, *p* < 0.05) groups [vs. the CUMS model group (13.60 ± 4.00 × 10^−3^%)]. The relative abundance of replication, and repair functions (Figure 6(B)) were increased in the fluoxetine (5.50 ± 4.00 × 10^−3^%, *p* < 0.01), TIV-L (5.60 ± 1.00 × 10^−3^%, *p* < 0.01), TIV-H (5.60 ± 1.00 × 10^−3^%, *p* < 0.01) [vs. the CUMS model group (5.10 ± 4.00 × 10^−3^%)]. In contrast, the relative abundance of infectious diseases (Figure 6(C)) were decreased in the fluoxetine (1.60 ± 4.00 × 10^−4^%, *p* < 0.01), TIV-L (1.60 ± 2.00 × 10^−4^%, *p* < 0.01), TIV-M (1.90 ± 5.00 × 10^−3^%, *p* < 0.05) and TIV-H (1.80 ± 3.00 × 10^−3^%, *p* < 0.05) groups [vs. the CUMS model group (2.20 ± 7.00 × 10^−3^%)].

### Effect of TIV on the expression of ZO-1 and occludin

**Figure 7.**
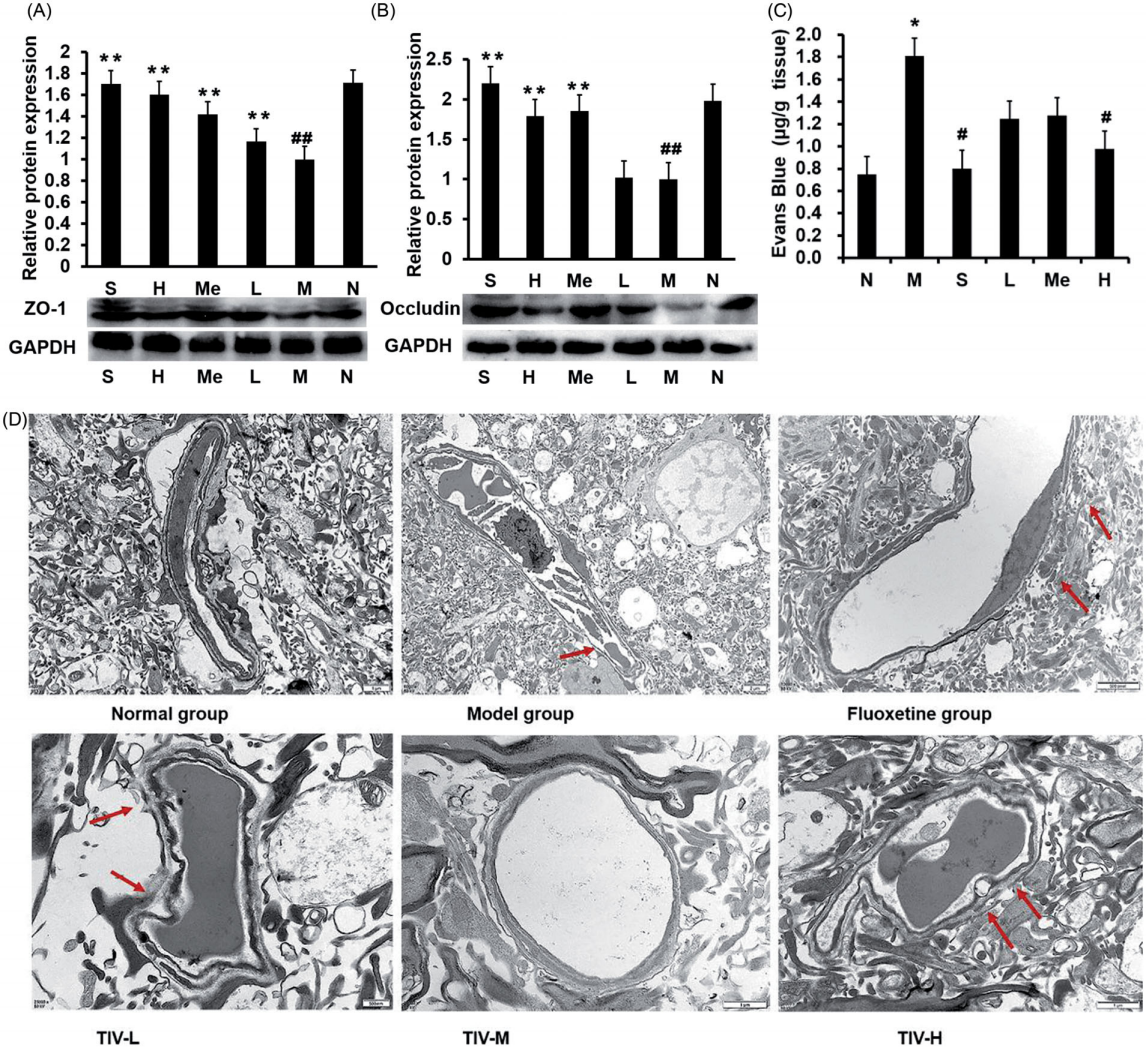
Comparison of BBB permeability and protein expression in TIV-treated CUMS mice and controls. (A) ZO-1 expression in TIV-treated CUMS mice. (B) Occludin expression in TIV-treated CUMS mice. (C) Changes in BBB permeability. (D) Observation of TJ ultrastructure changes in the BBB by transmission electron microscopy. The red arrow indicates a break or discontinuity in the basement membrane. Data are reported as mean ± SD. # *p* < 0.05 and ## *p* < 0.01 vs. the normal group. *p < 0.05 and **p < 0.01 vs. the model group. N: normal; M: model; S: fluoxetine; L: TIV-L; Me: TIV-M; H: TIV-H (*n* = 3 per group).

The expression of ZO-1 and occludin in the TJ was measured to clarify the mechanism of increased BBB permeability in mice after CUMS modelling. As shown in Figure 7(A,B), the expression of ZO-1 and occludin in the normal group was significantly higher than that of the model group (1.71-fold, *p* < 0.01; 1.98-fold, *p* < 0.01). TIV up-regulated the expression of ZO-1 (1.42-fold, *p* < 0.01; 1.60-fold, *p* < 0.01 and 1.71-fold, *p* < 0.01 for TIV-L, TIV-M and TIV-H, respectively) and occludin (1.79-fold, *p* < 0.01 for TIV-M and 2.20-fold, *p* < 0.01 for TIV-H) in the TJ in mice.

### Effect of TIV on the permeability and structure of the BBB

The BBB is essential for maintaining homeostasis in the brain (Jing et al. 2020). Increased BBB permeability destabilises the brain’s internal environment, resulting in CNS damage and disease (Wang et al. 2014). We explored the effect of TIV on BBB permeability and structure in depressed mice. Figure 7(C) shows EB content, a measurement of BBB permeability, in mouse brains. EB content increased, indicating increased BBB permeability in the brains of mice from the CUMS model group compared to normal controls (1.81 ± 0.33 vs. 0.75 ± 0.18 μg/g, *p* < 0.05). The EB content was reduced in the brains of mice in the TIV-H group (0.77 ± 0.30 μg/g, *p* < 0.05) and the fluoxetine (0.80 ± 0.22 μg/g, *p* < 0.05) (vs. the CUMS group). Figure 7(D) shows the changes in the morphological structure of the BBB. Compared with the normal group, the microvascular endothelial cell layer of the BBB in the model group was almost normal under the electron microscope, but the basement membrane was thin with some broken areas. However, the fluoxetine group and each TIV-L and TIV-H group exhibited improved TJ ultrastructure changes in the BBB structure compared with the model group.

## Discussion

Successful establishment of the CUMS model is critical for this study. The CUMS model was established by applying chronic, unpredictable, mildly stimulating stressors for 4 weeks (Xu et al. 2015). The CUMS rats showed bodyweight loss, prolonged immobility, and decreased sugar consumption, all of which indicate the model’s suitability for observing the effects of TIV on experimental CUMS in rats.

Decreased ZO-1 expression increases BBB permeability, and serine/threonine dephosphorylation of occludin affects TJ structure, causing BBB dysfunction (Nighot et al. 2017). Our studies showed that CUMS affected ZO-1 and occludin expression and changed the BBB structure, whereas TIV increased the expression of ZO-1 and occludin to reduce BBB permeability in depressed mice. TIV-L and TIV-H mice exhibited improved TJ ultrastructure changes in the BBB. Changes in TJ structure and function may be caused by an immune response, trauma, ischaemia, or hypoxia, which leads to increased permeability of BBB and brain damage (Feng et al. 2018; Yang et al. 2018).

In the gastrointestinal tract, the dominant bacterial species are divided into three phyla Bacteroidetes, Firmicutes (i.e., *Lactobacillus* spp.), and Actinobacteria, with other bacteria such as *Proteobacteria*, *Lactobacilli*, *Streptococci*, and *Escherichia coli* found in small numbers (Riaz Rajoka et al. 2017; Pascale et al. 2018). Consistent with the finding of previous reports, we found the CUMS mice microbiome becomes dominated by the Firmicutes (i.e., *Lactobacillus* spp.), Proteobacteria, and Bacteroidetes (i.e., *Bacteroides* spp.). Many intestinal microorganisms have a symbiotic relationship with the organism (Qin et al. 2010). Stressed mice also have been shown to have high populations of Firmicutes (i.e., *Lactobacillus* spp.), Bacteroidetes, Actinobacteria, and Proteobacteria (Rieder et al. 2017; Lach et al. 2018). In our study, TIV administration balanced the relative abundance of Firmicutes (i.e., *Lactobacillus* spp.), Bacteroidetes, and Proteobacteria in the mouse intestine. 16S rRNA sequencing showed that the CUMS model affects the composition and structure of the intestinal flora in mice, resulting in functional changes and physiological impacts. Changes in the composition and diversity of the gut microbiome lead to the production of various neurotransmitters, hormones, and metabolic byproducts that stimulate the vagus nerve and enteric nervous system (Nadeem et al. 2019).

When the intestinal tract of adult sterile mice was colonised with bacterial strains producing SCFAs, BBB permeability was normal and sodium butyrate increased in the intestine (Ochoa-Reparaz and Kasper 2016). Butyrate affects the expression of TJ-related proteins claudin-2, occludin, cingulin, ZO-1, and ZO-2 (Ploger et al. 2012). BBB permeability was reduced and TJ protein expression was increased when sterile mice were exposed to the faecal microbiota of pathogen-free donors (Zhang 2015). In these studies, the intestinal flora profoundly affected the function and structure of the BBB. In our study, the administration of TIV induced changes in the intestinal flora. A significant increase in the order phylum Bacteroidetes and a decrease in the phylum Firmicutes (i.e., *Lactobacillus* spp.) were demonstrated in depressive rats (Pascale et al. 2020). Consistent with previous reports, the relative abundance of Firmicutes (i.e., *Lactobacillus* spp.) and Bacteroidetes phyla were close to the normal group after TIV-L treatment the CUMS mice. These findings have significance for future research on the antidepressant effect of TIV on the intestinal flora. We speculate that TIV-L may protect the BBB by regulating metabolic products metabolised by the intestinal flora, affecting the expressions of ZO-1 and occludin.

The limitations to our study include (1) an absence of aseptic controls, (2) limited exploration of the antidepressant mechanism of TIV in the BBB-intestinal flora pathway, and (3) a small sample size. In future studies, changes in bacterial abundance caused by CUMS and BBB protection by metabolites, such as SCFAs, should be confirmed at the molecular level. These studies confirm the hypothesis that the BBB may be susceptible to changes in intestinal microflora.

## Conclusions

We speculate that TIV may enhance the predominance of Firmicutes (i.e., *Lactobacillus* spp.) and Bacteroidetes to regulate the structure and function of the intestinal flora and altering expression of ZO-1 and occludin, thus protecting the BBB to exert an antidepressant effect. This study may provide a theoretical basis for VJJ as a new drug to treat depression.

## References

[CIT0001] Biala G, Pekala K, Boguszewska-Czubara A, Michalak A, Kruk-Slomka M, Budzynska B. 2017. Behavioral and biochemical interaction between nicotine and chronic unpredictable mild stress in mice. Mol Neurobiol. 54(2):904–921. 26780460 10.1007/s12035-016-9701-0 PMC5310564

[CIT0002] Braniste V, Al-Asmakh M, Kowal C, Anuar F, Abbaspour A, Tóth M, Korecka A, Bakocevic N, Kundu P, Gulyás B, et al. 2014. The gut microbiota influences blood-brain barrier permeability in mice. Sci Transl Med. 6(263):263ra158. 10.1126/scitranslmed.3009759 PMC4396848 25411471

[CIT0003] Cipriani A, Furukawa TA, Salanti G, Chaimani A, Atkinson LZ, Ogawa Y, Leucht S, Ruhe HG, Turner EH, Higgins JPT, et al. 2018. Comparative efficacy and acceptability of 21 antidepressant drugs for the acute treatment of adults with major depressive disorder: a systematic review and network meta-analysis. The Lancet. 391(10128):1357–1366. 10.1016/S0140-6736(17)32802-7 PMC5889788 29477251

[CIT0004] Cryan J, Dinan T. 2015. Gut microbiota: microbiota and neuroimmune signalling-Metchnikoff to microglia. Nat Rev Gastroenterol Hepatol. 12(9):494–496. 26215386 10.1038/nrgastro.2015.127

[CIT0005] Deng L, Chen H, Wei N, Zhang Z, Wang G. 2019. The cardioprotective effect of dexmedetomidine on regional ischemia/reperfusion injury in type 2 diabetic rat hearts. Microvasc Res. 123:1–6. 30179598 10.1016/j.mvr.2018.08.006

[CIT0006] Di Marco A, Vignone D, Gonzalez Paz O, Fini I, Battista MR, Cellucci A, Bracacel E, Auciello G, Veneziano M, Khetarpal V, et al. 2020. Establishment of an *in vitro* human blood–brain barrier model derived from induced pluripotent stem cells and comparison to a porcine cell-based system. Cells. 9(4):994–922. 32316221 10.3390/cells9040994 PMC7226989

[CIT0007] Dong FW, Jiang HH, Yang L, Gong Y, Zi CT, Yang D, Ye CJ, Li H, Yang J, Nian Y, et al. 2018. Valepotriates from the roots and rhizomes of *Valeriana jatamansi* Jones as novel N-type calcium channel antagonists. Front Pharmacol. 9:1–9. 30150936 10.3389/fphar.2018.00885 PMC6099110

[CIT0008] Feng S, Zou L, Wang H, He R, Liu K, Zhu H. 2018. RhoA/ROCK-2 pathway inhibition and tight junction protein upregulation by catalpol suppresses lipopolysaccaride-induced disruption of blood–brain barrier permeability. Molecules. 23(9):2371– 2317. 30227623 10.3390/molecules23092371 PMC6225311

[CIT0009] Gao YB, Xiang LI, Wang SM, Zhao L, Yao BW. 2011. Application of Evans blue dye to the detection of permeability of BBB after microwave radiation. Milit Med Sci. 35:391–393.

[CIT0010] Gartlehner G, Morgan LC, Richard HA. 2012. P-1102 - Comparative effectiveness of second-generation antidepressants in the pharmacologic treatment of adult depression. Eur Psychiat. 27:1–1. 20704050

[CIT0011] Huang J, Zhang W, Fan R, Liu Z, Huang T, Li J, Du T, Xiong T. 2020. Composition and functional diversity of fecal bacterial community of wild boar, commercial pig and domestic native pig as revealed by 16S rRNA gene sequencing. Arch Microbiol. 202(4):843–857. 31894392 10.1007/s00203-019-01787-w

[CIT0012] Jing Y, Yang DX, Wang W, Yuan F, Chen H, Ding J, Geng Z, Tian HL. 2020. Aloin protects against blood-brain barrier damage after traumatic brain injury in mice. Neurosci Bull. 36(6):625–638. 32100248 10.1007/s12264-020-00471-0 PMC7270422

[CIT0013] Kealy J, Greene C, Campbell M. 2020. Blood-brain barrier regulation in psychiatric disorders. Neurosci Lett. 726:133664. 29966749 10.1016/j.neulet.2018.06.033

[CIT0014] Khan A-u, Gilani A-H. 2011. Pharmacological basis for the medicinal use of *Valeriana wallichii* in constipation. Lat Am J Pharm. 30(1):186–188.

[CIT0015] Kigerl KA, Hall JC, Wang L, Mo X, Yu Z, Popovich PG. 2016. Gut dysbiosis impairs recovery after spinal cord injury. J Exp Med. 213(12):2603–2620. 27810921 10.1084/jem.20151345 PMC5110012

[CIT0016] Lach G, Schellekens H, Dinan TG, Cryan JF. 2018. Anxiety, depression, and the microbiome: a role for gut peptides. Neurotherapeutics. 15(1):36–59. 29134359 10.1007/s13311-017-0585-0 PMC5794698

[CIT0017] Letchamo W, Ward W, Heard B, Heard D. 2004. Essential oil of *Valeriana officinalis* L. cultivars and their antimicrobial activity as influenced by harvesting time under commercial organic cultivation. J Agric Food Chem. 52(12):3915–3919. 15186117 10.1021/jf0353990

[CIT0018] Li J, Peng L, Huang HH, Cao H. 2009. Research progress of *in vitro* model building of blood–brain barrier. Acad J Guangdong Coll Pharm. 30:647–648.

[CIT0019] Li R. 2009. Extraction and analysis of flavonoids from *Valeriana jatamansi* and its anti-inflammatory and antibacterial activities [master’s thesis]. Jiangsu: Suzhou University.

[CIT0020] Li SZ. 1975. Compendium of materia mdica. Beijing: People's Medical Publishing House.

[CIT0021] Li Y, Wu L, Chen C, Wang L, Guo C, Zhao X, Zhao T, Wang X, Liu A, Yan Z. 2020. Serum metabolic profiling reveals the antidepressive effects of the total iridoids of *Valeriana jatamansi* Jones on chronic unpredictable mild stress mice. Front Pharmacol. 11:1–13. 32265710 10.3389/fphar.2020.00338 PMC7099651

[CIT0022] Lin S, Shen Y-H, Li H-L, Yang X-W, Chen T, Lu L-H, Huang Z-S, Liu R-H, Xu X-K, Zhang W-D, et al. 2009. Acylated iridoids with cytotoxicity from *Valeriana jatamansi*. J Nat Prod. 72(4):650–655. 19245261 10.1021/np800716f

[CIT0023] Nadeem I, Rahman MZ, Ad-Dab'bagh Y, Akhtar M. 2019. Effect of probiotic interventions on depressive symptoms: a narrative review evaluating systematic reviews. Psychiatry Clin Neurosci. 73(4):154–162. 30499231 10.1111/pcn.12804

[CIT0024] Nakagawasai O, Yamada K, Takahashi K, Odaira T, Sakuma W, Ishizawa D, Takahashi N, Onuma K, Hozumi C, Nemoto W, et al. 2020. Liver hydrolysate prevents depressive-like behavior in an animal model of colitis: involvement of hippocampal neurogenesis via the AMPK/BDNF pathway. Behav Brain Res. 390:112640. 32434062 10.1016/j.bbr.2020.112640

[CIT0025] Nighot PK, Leung L, Ma TY. 2017. Chloride channel ClC- 2 enhances intestinal epithelial tight junction barrier function via regulation of caveolin-1 and caveolar trafficking of occludin. Exp Cell Res. 352(1):113–122. 28161538 10.1016/j.yexcr.2017.01.024 PMC5328798

[CIT0026] Ochoa-Reparaz J, Kasper LH. 2016. The second brain: is the gut microbiota a link between obesity and central nervous system disorders? Curr Obes Rep. 5(1):51–64. 26865085 10.1007/s13679-016-0191-1 PMC4798912

[CIT0027] Pascale A, Marchesi N, Govoni S, Barbieri A. 2020. Targeting the microbiota in pharmacology of psychiatric disorders. Pharmacol Res. 157:104856. 32389857 10.1016/j.phrs.2020.104856

[CIT0028] Pascale A, Marchesi N, Marelli C, Coppola A, Luzi L, Govoni S, Giustina A, Gazzaruso C. 2018. Microbiota and metabolic diseases. Endocrine. 61(3):357–371. 29721802 10.1007/s12020-018-1605-5

[CIT0029] Perrech M, Dreher L, Rohn G, Stavrinou P, Krischek B, Toliat M, Goldbrunner R, Timmer M. 2019. Qualitative and quantitative analysis of IDH1 mutation in progressive gliomas by allele-specific qPCR and Western Blot Analysis. Technol Cancer Res Treat. 18:1533033819828396. 30943868 10.1177/1533033819828396 PMC6457076

[CIT0030] Ploger S, Stumpff F, Penner GB, Schulzke JD, Gabel G, Martens H, Shen Z, Gunzel D, Aschenbach JR. 2012. Microbial butyrate and its role for barrier function in the gastrointestinal tract. Ann N Y Acad Sci. 1258:52–59. 22731715 10.1111/j.1749-6632.2012.06553.x

[CIT0031] Prasad R, Naime M, Routray I, Mahmood A, Khan F, Ali S. 2010. *Valeriana jatamansi* partially reverses liver cirrhosis and tissue hyperproliferative response in rat. Methods Find Exp Clin Pharmacol. 32(10):713–719. 21225006 10.1358/mf.2010.32.10.1522224

[CIT0032] Qin J, Li R, Raes J, Arumugam M, Burgdorf KS, Manichanh C, Nielsen T, Pons N, Levenez F, Yamada T, Mende DR.; MetaHIT Consortium. 2010. A human gut microbial gene catalogue established by metagenomic sequencing. Nature. 464(7285):59–65. 20203603 10.1038/nature08821 PMC3779803

[CIT0033] Riaz Rajoka MS, Shi J, Mehwish HM, Zhu J, Li Q, Shao D, Huang Q, Yang H. 2017. Interaction between diet composition and gut microbiota and its impact on gastrointestinal tract health. Food Sci Human Wellness. 6(3):121–130.

[CIT0034] Rieder R, Wisniewski PJ, Alderman BL, Campbell SC. 2017. Microbes and mental health: a review. Brain Behav Immun. 66:9–17. 28131791 10.1016/j.bbi.2017.01.016

[CIT0035] Rook GA, Lowry CA, Raison CL. 2013. Microbial ‘Old Friends’, immunoregulation and stress resilience. Evol Med Public Health. 2013(1):46–64. 24481186 10.1093/emph/eot004 PMC3868387

[CIT0036] Sah SP, Mathela CS, Chopra K. 2010. Elucidation of possible mechanism of analgesic action of *Valeriana wallichii* DC chemotype (patchouli alcohol) in experimental animal models. Indian J Exp Biol. 48(3):289–293. 21046983

[CIT0037] Sherwin E, Sandhu KV, Dinan TG, Cryan JF. 2016. May the force be with you: the light and dark sides of the Microbiota-Gut-Brain Axis in Neuropsychiatry. CNS Drugs. 30(11):1019–1041. 27417321 10.1007/s40263-016-0370-3 PMC5078156

[CIT0038] Shi RR, Wang J, Yan XL, Hu JH, Gao ZP, Jia MX, Yu X. 2011. Effective mechanism of iridoid constituents of Zhizhuxiang (*Valeriana jatamans* Rhizome) in rats with irritable bowel syndrome. J Beijing Univ Tradit Chin Med. 36:1235–1238.

[CIT0039] Singh N, Gupta AP, Singh B, Kaul VK. 2006. Quantification of Valerenic acid in *Valeriana jatamansi* and *Valeriana officinalis* by HPTLC. Chroma. 63(3-4):209–213.

[CIT0040] Song H, Yu ZL, Yang MJ, Zhang T, Wang HY. 2018. Analysis of microbial abundance and community composition in esophagus and intestinal tract of wild veined rapa whelk (*Rapana venosa*) by 16S rRNA gene sequencing. J Gen Appl Microbiol. 64(4):158–166. 29643283 10.2323/jgam.2017.11.003

[CIT0041] Subhan F, Karim N, Gilani AH, Sewell RDE. 2010. Terpenoid content of *Valeriana wallichii* extracts and antidepressant-like response profiles. Phytother Res. 24(5):686–691. 19943315 10.1002/ptr.2980

[CIT0042] Tan YZ. 2019. Chemical constituents from *Valeriana jatamansi* and their antitumor activities. Chin Tradit Pat Med. 41:572–576.

[CIT0043] Tang Y, Liu X, Yu B. 2002. Iridoids from the rhizomes and roots of *Valeriana jatamansi*. J Nat Prod. 65(12):1949–1952. 12502349 10.1021/np0203335

[CIT0044] Tao SY. 2017. The therapeutic effect of *Valeriana jatamasi* component ZXY on IBS model rats and its peripheral mechanism on blood–gut interaction [master’s thesis]. [Beijing: Beijing University of Chinese Medicine].

[CIT0045] Tikiyani V, Babu K. 2019. Claudins in the brain: unconventional functions in neurons. Traffic. 20(11):807–814. 31418988 10.1111/tra.12685

[CIT0046] Tobiansky DJ, Kachkovski GV, Enos RT, Schmidt KL, Murphy EA, Soma KK. 2020. Sucrose consumption alters steroid and dopamine signalling in the female rat brain. J Endocrinol. 245(2):231–246. 32112695 10.1530/JOE-19-0386

[CIT0047] Wang L, Sun Y, Zhao T, Li Y, Zhao X, Zhang L, Wu L, Zhang L, Zhang T, Wei G, et al. 2020. Antidepressant effects and mechanisms of the total iridoids of *Valeriana jatamansi* on the Brain-Gut Axis. Planta Med. 86(3):172–179. 31801162 10.1055/a-1068-9686

[CIT0048] Wang LP, Jianfan F. Hukaili. 2014. Progress in regulation effect of aromatic refreshing traditional Chinese medicine on BBB permeability and its mechanism. J Tradit Chin Med. 39:949–954. 24956831

[CIT0050] Xu KK, Li SH, Zuo CY, Chen CY, Zhang RT, Lan M, Lin Y, Zhang TE, Yan ZY. 2014. Content determination of chlorovaltrate and valjatrate B in total iridoids of *Valerianae jatamansi* Rhizoma et radix. Chin J Exp Tradit Med Formul. 20(16):64–66.

[CIT0051] Xu KK, Lin Y, Zhang R, Lan M, Chen C, Li S, Zuo C, Chen C, Zhang T, Yan Z. 2015. Evaluation of safety of iridoids rich fraction from *Valeriana jatamansi* Jones: acute and sub-chronic toxicity study in mice and rats. J Ethnopharmacol. 172:386–394. 26164073 10.1016/j.jep.2015.06.046

[CIT0052] Yang Y, Kimura-Ohba S, Thompson JF, Salayandia VM, Cosse M, Raz L, Jalal FY, Rosenberg GA. 2018. Vascular tight junction disruption and angiogenesis in spontaneously hypertensive rat with neuroinflammatory white matter injury. Neurobiol Dis. 114:95–110. 29486300 10.1016/j.nbd.2018.02.012 PMC5891378

[CIT0053] Zhang J, Takahashi HK, Liu K, Wake H, Liu R, Maruo T, Date I, Yoshino T, Ohtsuka A, Mori S, et al. 2011. Anti-high mobility group box-1 monoclonal antibody protects the blood–brain barrier from ischemia-induced disruption in rats. Stroke. 42(5):1420–1428. 21474801 10.1161/STROKEAHA.110.598334

[CIT0054] Zhang MZ. 2015. The effect of intestinal microbiota on the permeability of blood–brain barrier in mice. Prog Physiol Sci. 46(2):132–132.

[CIT0055] Zhu J, Xu K, Zhang X, Cao J, Jia Z, Yang R, Ma C, Chen C, Zhang T, Yan Z. 2016. Studies on the regulation of lipid metabolism and its mechanism of the iridoids rich fraction in *Valeriana jatamansi* Jones. Biomed Pharmacother. 84:1891–1898. 27832992 10.1016/j.biopha.2016.10.099

