# Correction

**DOI:** 10.1080/13880209.2023.2235925

**Published:** 2023-07-14

**Article title:** Antidepressant effects of total iridoids of *Valeriana jatamansi* via the intestinal flora-blood–brain barrier pathway

**Authors:** Zhang, L., Wang, L., Huang, L., Zhao, Y., Ding, H., Li, B., Wen, L., Xiong, W., Liu, Y., Zhang, T., Zhang, L., Wu, L., Xu, Q., Fan, Y., Wei, G., Yin, Q., Chen, Y., Zhang, T., & Yan, Z.

**Journal:** *Pharmaceutical Biology*

**Bibliometrics:** Volume 59, Number 01, pages 910–919

**DOI:** https://doi.org/10.1080/13880209.2021.1944222

In the version of this article initially published, there was a mistake in the images of part labels (A) and (B) of Figure 7.

The images of part labels (A) and (B) of Figure 7 are now corrected and the article has been republished online along with a minor correction where the author Zhiyong Yan’s contact details have been added.

